# Genome Sequence of *Magnetospirillum magnetotacticum* Strain MS-1

**DOI:** 10.1128/genomeA.00233-15

**Published:** 2015-04-02

**Authors:** Matthew D. Smalley, Georgi K. Marinov, L. Elizabeth Bertani, Gilberto DeSalvo

**Affiliations:** Division of Biology and Biological Engineering, California Institute of Technology, Pasadena, California, USA

## Abstract

Here, we report the genome sequence of *Magnetospirillum magnetotacticum* strain MS-1, which consists of of 36 contigs and 4,136 protein-coding genes.

## GENOME ANNOUNCEMENT

*Magnetospirillum magnetotacticum* strain MS-1 (1) is an alphaproteobacterial representative of magnetotactic bacteria. Magnetotactic bacteria are a unique group of prokaryotes characterized by their ability to orient themselves along the lines of a magnetic field (magnetotaxis), conferred by the presence of specialized organelles called magnetosomes (2, 3). Magnetosomes are a rare example of a lipid-bounded intracellular organelle in prokaryotes and are of great interest both from a purely biological perspective and because of their numerous potential technological applications (4, 5).

An assembly of the MS-1 genome was previously deposited in GenBank (assembly ID GCA_000166875.1). However, the annotation of that assembly revealed that it is heavily contaminated with sequences from an unidentified Methylobacter strain. It contains two sets of rRNAs, one of which is a best match to Methylobacterium rRNAs, and >60% of the predicted open reading frames (ORFs) also had a best match in the genome of one of the sequenced Methylobacterium strains. We therefore carried out *de novo* whole-genome sequencing of the MS-1 strain. We generated ~1,000× coverage of the MS-1 genome using 2 × 150-bp Illumina HiSeq reads, with an average fragment length of ~700 bp. The reads were error corrected using BayesHammer (6) and assembled using SPAdes (version 3.1.1) (7), IDBA (version 1.1.1) (8), and Edena (version 3.131028) (9). Contigs with a coverage of ≤200× and length ≤200 bp were filtered out, and the assemblies were merged with CISA (version 1.3) (10). After filtering out potential contaminants (i.e., contigs that were close matches to the human and other eukaryotic genome sequences), the final assembly consists of 4,523,935 bp in 36 contigs, with an *N*_50_ of 541,957 and *N*_90_ of 102,810. The largest contig is 1,344,907 bp in length, and the G+C content is 63.5%.

The annotation of protein-coding genes was carried out using RAST (11). Ribosomal RNAs were annotated using RNAmmer 1.2 (12), and tRNAscan-SE (version 1.21) (13) was used to find tRNA genes. Additional noncoding RNA genes and elements were annotated using Infernal (version 1.1.1) (14) and the Rfam database (version 12.0) (15).

A total of 4,136 protein-coding genes, 48 tRNAs, and a single set of rRNAs were predicted. The vast majority of the genes have their best BLAST match in the genomes of related magnetotactic bacteria, such as Magnetospirillum sp. strain AMB-1, indicating the absence of contamination in the assembly.

### Nucleotide sequence accession numbers.

This whole-genome shotgun project has been deposited in GenBank under the accession no. JXSL00000000. The version described in this paper is the first version, JXSL01000000. The raw sequencing reads are available from the Short Read Archive under the accession no. SRR1765663.
